# 2578. Trends in Pathogens and Antibiotic Treatment in Children with Cystic Fibrosis in 2018 and 2022: A Retrospective Multicenter Study

**DOI:** 10.1093/ofid/ofad500.2194

**Published:** 2023-11-27

**Authors:** Dayna Stout, Yaron Fireizen, Timothy Vigers, Mohamoud Ahmed, Kathryn Akong, Julie Ryu, Andrea Hahn, Anastassios Koumbourlis, Pornchai Tirakitsoontorn, Antonio C Arrieta, Elizabeth Burgener, Elinor Towler, Allison Keck, Scott Sagel, John S Bradley

**Affiliations:** Rady Children's Hospital San Diego, San Diego, CA; Rady children's hospital/University of California San Diego, La Jolla, California; University of Colorado Anschutz Medical Campus, Aurora, Colorado; University of Colorado Anschutz Medical Campus, Aurora, Colorado; Rady children's hospital/University of California San Diego, La Jolla, California; Rady children's hospital/University of California San Diego, La Jolla, California; Children's National Health System, Washington, DC; Children's National Health System, Washington, DC; Children’s Hospital of Orange County/University of California Irvine, Orange, California; Children's Hospital of Orange County, Orange, California, USA, Orange, California; Center for Excellence in Pulmonary Biology, Dept of Pediatrics, Stanford University School of Medicine, Stanford, California; Children's Hospital Colorado, Denver, Colorado; Children's Hospital Colorado, Denver, Colorado; University of Colorado, Anschutz Medical Campus, Aurora, Colorado; University of San Diego School of Medicine, Rady Children's Hospital, San Deigo, California

## Abstract

**Background:**

The new Cystic Fibrosis (CF) transmembrane conductance regulator modulator therapy combination (elexacaftor/tezacaftor/ivacaftor, ETI) has significantly improved the quality of life of children with CF and decreased the rate of pulmonary exacerbations (PEx). The focus of our study was to examine the infection characteristics of PEx in children with CF before the availability of ETI (2018) and following ETI approval and during emergence from COVID-19 (2022).

**Methods:**

We conducted a multicenter (5 sites) retrospective review collecting data on children with CF over 12-month periods in 2018 and in 2022 who were hospitalized for PEx and treated with IV antibiotics. We analyzed the number of PEx, bacterial and fungal organisms identified by CF respiratory cultures and IV antibiotics used empirically at hospital admission.

**Results:**

The number of hospitalizations for PEx decreased from 414 in 2018 to 163 in 2022. The most common organisms identified in all centers were Pseudomonas aeruginosa (Pa), Staphylococcus aureus (Sa) and Candida species, with no apparent overall difference in the relative percentages of pathogens isolated between 2018 and 2022 (figure 1), although fewer children were infected with methicillin-resistant S. aureus (MRSA) in 2022 (2.7%) than in 2018 (10%) (p=0.014). There was variability in the type of antipseudomonal agents and antistaphylococcal agents used empirically both within and between the different centers (figure 2).

**Figure:**
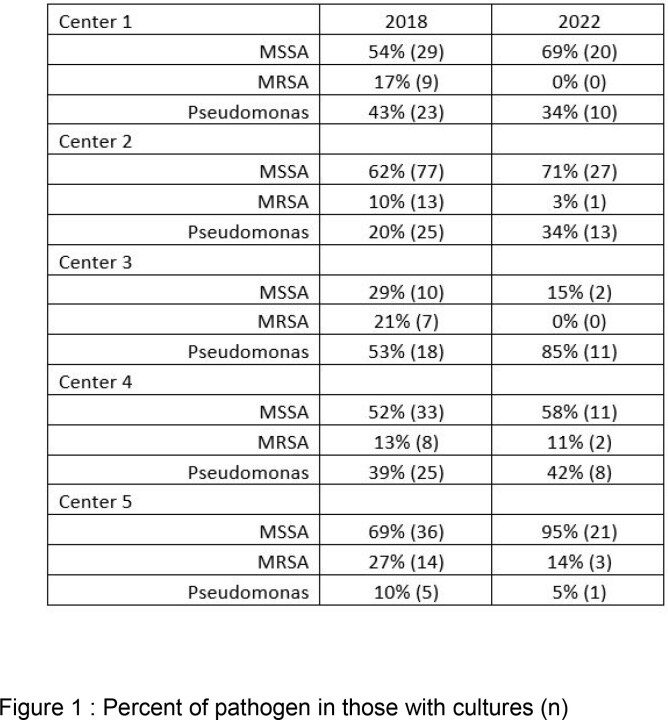

**Figure:**
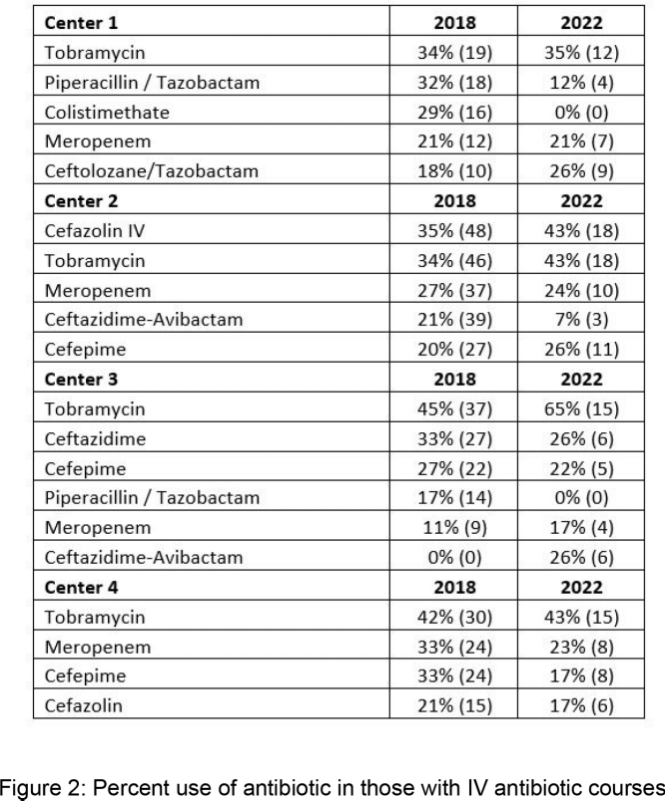

**Conclusion:**

Pathogens isolated from respiratory culture associated with PEx demonstrated similar rates of isolation of pathogens in the 2 time periods, consistent with historical CF pathogen isolation. The decreased number of PEx in 2022 compared to 2018 is consistent with the widespread use of ETI. The variability of usage antipseudomonal agents between centers and between years of data collection requires further investigation.

**Disclosures:**

**Andrea Hahn, MD, MS**, TGV Dx: Advisor/Consultant **Antonio C. Arrieta, MD, FIDSA, FPIDS**, Astellas Pharma Global Development, Inc.: Advisor/Consultant|Astellas Pharma Global Development, Inc.: Grant/Research Support|Astellas Pharma Global Development, Inc.: Honoraria|Cumberland Pharmaceutical: Grant/Research Support|IDbyDNA: Advisor/Consultant|IDbyDNA: Grant/Research Support|Melinta: Grant/Research Support|Merck: Advisor/Consultant|Merck: Grant/Research Support|Nabriva: Grant/Research Support|Paratek Pharmaceuticals: Grant/Research Support|Pfizer, Inc: Advisor/Consultant|Pfizer, Inc: Grant/Research Support|Roche/Genentech: Grant/Research Support|The Medicine Company: Grant/Research Support

